# The Effect of Mold Flux Wetting Conditions with Varying Crucible Materials on Crystallization

**DOI:** 10.3390/ma18051174

**Published:** 2025-03-06

**Authors:** Muhammad Anwarul Nazim, Arezoo Emdadi, Todd Sander, Ronald O’Malley

**Affiliations:** Materials Science and Engineering, Missouri University of Science and Technology, 1400 N. Bishop Ave., Rolla, MO 65409-0340, USAomalleyr@mst.edu (R.O.)

**Keywords:** mold fluxes, nucleation, crystallization, crucible materials, wetting conditions, DSC, CLSM

## Abstract

Understanding mold flux crystallization is essential for assessing heat transfer during steel casting. The complexity of the mold gap presents challenges in identifying the optimal testing method and nucleation type. This study investigates how variations in wetting properties influence nucleation dynamics, in particular the wetting behaviors of mold flux in platinum and graphite crucibles and how they affect crystallization temperatures and solidification mechanisms. Advanced analytical techniques, including confocal laser scanning microscopy (CLSM), and differential scanning calorimetry (DSC) were employed to analyze nucleation under different conditions, with calibration using synthetic slag, Li_2_SO_4_, and thermodynamic equilibrium simulations. The findings highlight the crucial role of crucible materials in modifying nucleation energy barriers and undercooling requirements. These insights enhance the understanding of mold flux behavior, contributing to the refinement of testing methodologies and the optimization of heat transfer and solidification processes in continuous casting.

## 1. Introduction

Mold flux is a multi-component oxyfluoride powder that is employed on top of liquid steel during continuous casting process. The mold powder melts when heated by the liquid steel pool and penetrates between the solidifying steel shell and the mold as casting occurs. The initial slag film that forms in the mold gap is mostly glassy due to the high cooling rates as it quenches against the cold mold wall. Over time, however, the fraction of crystallized phases in the slag film progressively increases as the glassy layer undergoes devitrification [1]. Crystallization, being one of the most pivotal factors for controlling heat transfer, plays a crucial role in steel casting. Alterations in its characteristics can lead to non-uniform horizontal heat transfer and suboptimal lubrication, potentially compromising process efficiency and product quality [2].

The study of mold flux crystallization kinetics provides insights into the crystalline phase fraction as a function of time and temperature. It also reveals the crystallization mechanisms of mold fluxes and helps in understanding variations in crystallization behavior across different flux systems [3]. Sarkar and Li [1] conducted a comprehensive literature review on isothermal and non-isothermal crystallization kinetics studies of mold fluxes. Differential scanning calorimetry (DSC) and confocal laser scanning microscopy (CLSM) are widely used thermo-analytical techniques for investigating crystallization kinetics. As reported in the literature [1], platinum pans or crucibles are commonly employed in the DSC testing of mold fluxes due to their chemical inertness and high melting point.

Platinum is a precious and expensive crucible material. In some cases, especially for mold flux examinations, destructive post-processing for specimen characterization is required, i.e., crucibles are cut and destroyed since mold flux strongly adheres to the inner wall of the crucible. Also, when the crucible is reused, it goes through a cleaning process that involves harsh chemicals, which can lead to safety and cost-related concerns. However, graphite is an inexpensive and abundant high-temperature material. Under conditions of low oxygen potential, graphite demonstrates chemical stability and a high level of resistance to thermal shock. Furthermore, it is a relatively soft material, facilitating machining into a various shapes and configurations [4]. Although graphite tends to oxidize at high temperatures in air, it can still be employed as a crucible material in advanced thermo-analytical techniques such as DSC and CLSM, as a neutral environment is ensured with the purging of high-purity inert gases. Moreover, graphite is an integral part of most mold flux systems. Graphite is the principal component used to control the melting rate of the flux. It is important to note that mold flux wetting behavior varies with the substrate or crucible material with which it is in contact when tested using advanced thermo-analytical techniques.

**Figure 1:**
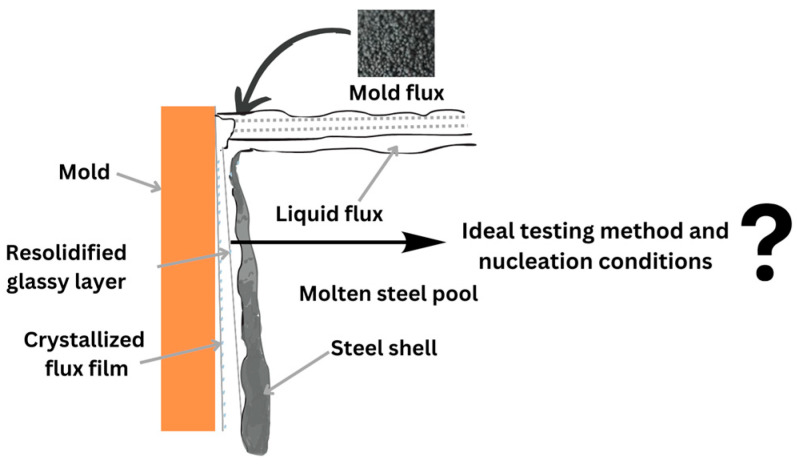
A schematic of the continuous casting process (not to scale), illustrating the complex mold gap region, where ideal testing methods and nucleation conditions are not well established.

Wettability describes how well a liquid can spread across a solid surface, assessed by the angle of wetting formed between the substrate and the tangent drawn at the triple point. Wetting is quantified using the contact angle, denoted *θ*. This angle forms where the substate, liquid, and gas interfaces meet, reflecting the balance of surface tension forces acting on the liquid droplet [5]. A contact angle of 180° signifies complete non-wetting, while, practically, a solid is considered non-wetting if the contact angle exceeds 90° [6]. Conversely, complete wetting occurs when the contact angle is 0°, allowing the liquid to spread spontaneously across the solid surface [5]. Standardized tests, such as DSC and CLSM, provide data on the onset of crystallization. However, in the complex environment of the mold gap during continuous casting, the wetting conditions among the mold, steel, and mold flux remain unclear. Numerous studies [7,8,9,10,11,12,13] have explored the wetting characteristics of mold flux droplets on steel substrates using sessile drop tests. These investigations primarily focused on the effects of compositional changes in slag and steel on wettability, arguing that the initial wettability of slag on steel significantly influences slag entrapment during the casting process and, consequently, the surface quality of the final slab. However, the literature reveals a notable knowledge gap regarding the impact of mold flux wetting behavior on copper molds and its role in crystallization, which in turn affects interfacial heat transfer during the casting process, as nucleation usually occurs on the mold wall side of the mold gap as illustrated in Figure 1.

Kromhout [14] reported in the literature that pumping during oscillation forces the liquid slag to spread on the copper mold surface. The interface adhering is non-wetting in that case. However, the conditions during casting seems to be opposite to this claim and require additional investigation. Park and Sohn [15] examined interfacial heat transfer between mold flux and a copper disc mold simulator (CDMS) as a function of the average waviness of the flux disc, where the liquid slag was poured into the concentric disc groove of the CDMS. Their study did not address the wettability of mold flux on the copper mold and the nature of the environment.

**Figure 2:**
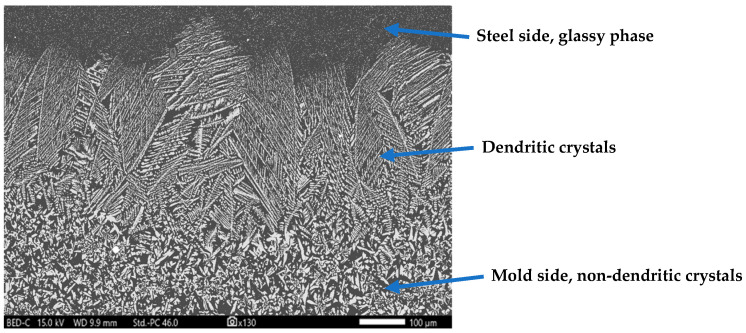
The micrograph of the mold flux film cross-section obtained through scanning electron microscopy (SEM). Courtesy: US Steel Research and Technology Center, PA, USA.

Apart from the copper disc mold simulator, a lab-scale mold simulator is another tool for understanding heat transfer during steel casting, particularly concerning the evolution of the flux film between the mold and cast steel. The literature offers a broad range of research on mold simulators [16,17,18,19,20,21,22,23,24,25,26], covering topics such as mold flux crystallization, heat transfer through mold flux, lubrication of mold flux, and interfacial thermal resistances in continuous casting of various steel grades. Recently, Nazim et al. [27] have implemented a novel approach to an optical fiber-instrumented mold simulator that can be further used to enhance the understanding of heat transfer through mold flux. According to the study, high-resolution optical fiber-based distributed temperature sensors can capture transient heat transfer phenomena. Figure 2 shows a micrograph of the mold flux film’s cross-section recovered from a mold simulator experiment. The flux film contains two component layers: a crystallite layer, which forms from the devitrification of the glassy layer and is non-dendritic in appearance, and a dendritic layer that grows against the heat transfer and is close to the steel side. These dendrites may nucleate heterogeneously from the pre-existing non-dendritic crystals, as supported by a previous study [28]. The key challenge lies in determining the testing methodology that best represents this environment and whether nucleation in the system is primarily heterogeneous or homogeneous. Another important consideration is the nature of this environment, and whether the mold wall contributes to wetting for nucleation, though the exact answer remains unclear.

To the best of our knowledge, no study has been performed to date on the effect of crucible material’s wettability on crystal nucleation in continuous casting mold flux. Novak et al. [29] investigated the wetting behavior of graphite and platinum substrates at 1550 °C with borate oxide glass systems containing B_2_O_3_ with an average basicity of 0.21. This study showed that contamination occurred in the oxide glass specimens as the graphite substrate wetted them, due to high adhesion and the formation of reaction products at the interface. However, continuous casting mold flux has a higher basicity, is silicate-based, and is utilized in different applications than the borate glass system. Additionally, the operating temperature of mold flux is significantly lower, and its wetting behavior with graphite and platinum appears to be the opposite of what was observed in the present study due to the formation of boron carbide.

This paper aims to investigate the influence of crucible wetting behavior on crystallization of an industrial-grade mold flux. Thermo-analytical techniques, including DSC and CLSM, were used to quantify the onset of primary crystallization of the flux through non-isothermal testing at various cooling rates. The flux specimens were characterized using scanning electron microscopy coupled with energy dispersive X-ray spectroscopy (SEM-EDS) and X-ray diffraction (XRD). Additional calibrations were conducted using gold and Li_2_SO_4_, followed by thermodynamic equilibrium simulations in Factsage^®^ to assess potential reaction with graphite. SEM-EDS analysis was performed post-mortem at the interface to assess potential reactions between the mold flux specimen and the graphite crucible wall. This study aims to enhance understanding of how wetting versus non-wetting affects crystal nucleation in mold flux, depending on whether platinum or graphite crucibles are used. The findings are of practical significance, as carbon or graphite is an essential component in mold flux design. Moreover, this manuscript may pave the way for future investigations into the influence of wetting characteristics on mold flux in the complex environment of the meniscus during continuous casting of steels.

## 2. Materials and Methods

### 2.1. Specimen Preparation

The CaO-SiO_2_-based mold flux used in this study was obtained from a mold flux manufacturing company. The flux is used to cast medium carbon, hyper-peritectic steel grades in a thin slab caster. Table 1 and Table 2 tabulate the flux’s chemical composition and physical properties, respectively. A specimen size of 60 mg was maintained for each experiment in both DSC and CLSM. Raw mold flux powder was calcined at 725 °C for 5 h in air, then pre-melted in a resistance furnace at 1300 °C. The molten flux was subsequently poured and rapidly quenched between two room-temperature copper plates. This process promotes the oxidation of the flux powder and removes volatile carbon and carbonaceous elements. The resulting glassy specimens were later used for crystallization studies in DSC and CLSM.

### 2.2. Test Procedures and Methods

#### 2.2.1. Crystallization Temperature Measurements Using Differential Scanning Calorimetry (DSC)

The DSC experiments were performed using a Thermal Gravimetric Analyzer/Differential Scanning Calorimeter (TGA-DSC), model Netzsch 449 F5 Jupiter (Netzsch-Gerätebau GmbH, Selb, Germany), with argon as the purge gas. Before testing, the setup was calibrated using pure substances (indium, tin, aluminum, bismuth, and gold) for both temperature and sensitivity calibration. A new baseline was established for each specific heating and cooling rate using empty crucibles, both graphite and platinum. After calibration, 60 mg of glassy specimens were heated to 1300 °C at a rate of 20 °C/min and held for 2 min for volatilization, followed by continuous cooling to room temperature at various rates of 5, 10, 15, 20, 25, and 30 °C/min, using platinum and graphite crucibles separately. The controls ensured that the test specimen and the reference temperatures were maintained the same throughout the experiment. The tests were conducted in an inert environment with an argon flow of 50 mL/min. The platinum pans used in the tests were 5 mm long with an inner diameter of 5 mm, while the graphite crucibles were 7 mm long and had a similar inner diameter. High-purity platinum pans were sourced from an external vendor, while graphite crucibles were machined from high-purity graphite rods.

#### 2.2.2. Observation of Onset of Crystallization Using Confocal Laser Scanning Microscopy (CLSM)

A 60 mg glassy flux specimen was placed in a graphite crucible, and the experiment was conducted in the melting stage using a Lasertec VL2000DX-SVF17SP High-Temperature CLSM system (Yonekura Mfg. Co. Ltd., Osaka, Japan) in an ultra-high-purity argon environment. Details of the experimental apparatus have been discussed elsewhere [30]. The thermal cycle in the CLSM was identical to that of the DSC, except for testing only one cooling rate (20 °C/min). The heating and holding phases were the same, with heating provided by light emitted from a halogen lamp. The test aimed to observe in situ crystallization on the flux surface within the graphite crucible during non-isothermal experiments at a cooling rate of 20 °C/min. The crystallization process was monitored and video recorded at a scanning rate of 60 frames per second. The specimen’s image was captured based on contrast differences produced by the scanning laser, operating within the ultraviolet wavelength range.

#### 2.2.3. X-Ray Diffraction for Phase Detection

XRD analysis is widely used to identify crystals formed during crystallization processes. Post-experiment specimens from the graphite crucibles were taken from the CLSM experiment, crushed into powder, and subjected to XRD analysis. Following the same thermal cycle as in the CLSM experiment, the specimen was cooled slowly to 1000 °C at a rate of 5 °C/min; this temperature is below the primary crystallization temperature, allowing sufficient time for primary crystals to nucleate. The specimen was then rapidly quenched to room temperature at a 2000 °C/min cooling rate. This process ensured that the formed primary crystal phase was retained and detectable using SEM and XRD. The XRD analysis was conducted using a Panalytical X’Pert Multi-Purpose Diffractometer (Malvern Panalytical B.V., Almelo, The Netherlands) with a 1.8 kW sealed copper X-ray source. The resulting peak list was compared with the Inorganic Crystal Structure Database (ICSD) data. XRD was also used to characterize the crystal phase of a common standard (Li_2_SO_4_) cooled from the melt.

#### 2.2.4. Computational Thermodynamic Analysis

Thermodynamic simulations were performed to assess the potential reactions between carbon or platinum and the common standard, Li_2_SO_4_. The equilibrium phases were calculated by simulating the reaction of 1 g of carbon or platinum with Li_2_SO_4_ using the Equilib module in FactSage^®^ 8.3 (Thermfact Ltd. and GTT Technologies, Montreal, QC, Canada/Aachen, Germany). The FToxid and FTmisc databases were utilized for the simulation. Phase stability diagrams were generated for a temperature range from 1000 °C to 0 °C.

#### 2.2.5. Calibration of Graphite Crucible Using Synthetic Slag, Pure Gold, and Li_2_SO_4_

The graphite crucible was calibrated using a gold standard in DSC with a moderate cooling rate of 20 °C/min. Additionally, a synthetic slag was tested in both graphite and platinum crucibles under the same cooling rate in DSC to evaluate the reactivity between the amphoteric oxide (FeO/Fe_2_O_3_ and MnO) components in the mold flux and the inner wall of the graphite crucible. Furthermore, a common standard, Li_2_SO_4_, was tested in both crucibles using the same procedure for calibration purposes.

#### 2.2.6. Elemental Map Using Scanning Electron Microscopy Coupled with Energy Dispersive X-Ray Spectroscopy (SEM-EDS)

SEM was used to further investigate the precipitated crystals. Additionally, SEM-EDS was employed for compositional analysis of the post-CLSM specimens at room temperature and the post-experiment DSC mold flux specimen. It was also used to examine the interface of the graphite-run DSC specimen in a TESCAN VEGA SEM (Bruker Nano GmbH, Berlin, Germany). The post-experiment flux specimen was mounted in epoxy and polished to 0.5 microns. The polished surface was then coated with a gold–palladium (Au–Pd) layer to enhance electrical conductivity using a Denton sputter coater, set to 8 mA for 45 s. A copper tape was applied to create an electrical path from the bottom to the top (surface) of the specimen for SEM analysis. This analysis helps determine whether carbon particles from the graphite crucible infiltrated the mold flux specimen, providing insights into potential interactions at the interface between the mold flux and the graphite inner wall.

## 3. Results and Discussion

### 3.1. Crucible Material-Dependent Wetting Characteristics

Mold flux in contact with the mold wall/substrate is considered to be a surface-assisted heterogeneous nucleation process, owing to its lower energy requirement across the bonding interface than that of homogeneous nucleation [31]. In heterogeneous nucleation, the wetting/contact angle (*θ*) characterizes the interaction between the newly formed phase and the substrate/mold wall, by reducing the energy needed. The free energy required for heterogeneous nucleation is equal to the product of homogeneous nucleation and a function of the contact/wetting angle (*θ*), as follows [31]:

Δ*G_heterogeneous_* = Δ*G_homogeneous_* × *f*(*θ*) (1)

where *f*(*θ*) = $\frac{2-3cos\theta +{cos}^{3}\theta }{4}$ and *θ* is the wetting/contact angle.

The crucible–mold flux system best represents the gas–liquid–mold system. The classical Young’s equation defines the equilibrium contact angle at the interface of the system, which is determined by the balance of surface energies, as follows:

γ_(*G–M*)_ = γ_(*G–L*)_ + γ_(*L–M*)_
*cosθ* (2)

where γ = surface tension, and (*G–M*), (*G–L*), and (*L–M*) represent the gas–mold, gas–liquid and liquid–mold interfaces, respectively.

**Figure 3:**
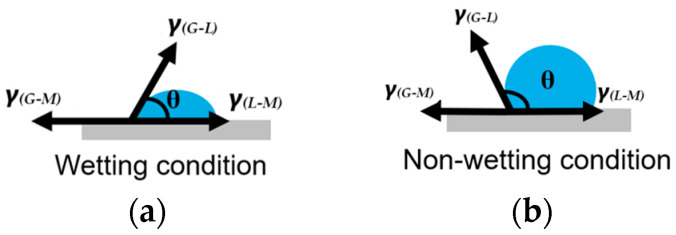
Schematics of (**a**) wetting and (**b**) non-wetting conditions of mold flux in contact with mold wall.

**Figure 4:**
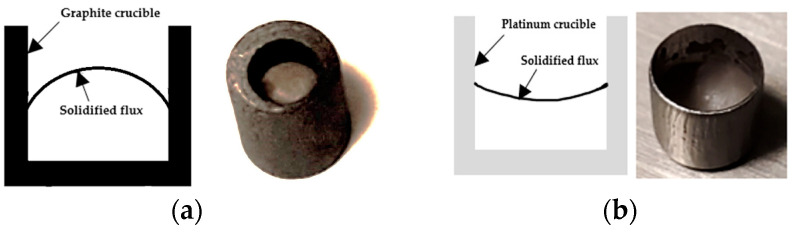
Schematics of the section slice of the flux-crucible appearance right after the DSC test in (**a**) a graphite crucible and (**b**) a platinum crucible.

Figure 3 illustrates the schematics of wetting and non-wetting conditions of a droplet of a material (in this case, mold flux) that solidify from a melt in contact with different crucible materials/mold wall. The cross-section of the post-experiment DSC solidified mold flux specimens revealed concave and convex surface shapes for platinum and graphite crucibles, respectively. Figure 4 shows the post DSC experiment mold flux in graphite and platinum crucibles and the schematics of the surface shape of solidified flux specimens in graphite and platinum crucibles from a cross-sectional view.

The mold flux exhibited non-wetting behavior with a large contact angle with the graphite crucible, whereas it wetted the platinum crucible with a small contact angle, resulting in opposite surface orientations.

### 3.2. Measurements of Crystallization Temperature Depending on Crucible Materials in DSC

**Figure 5:**
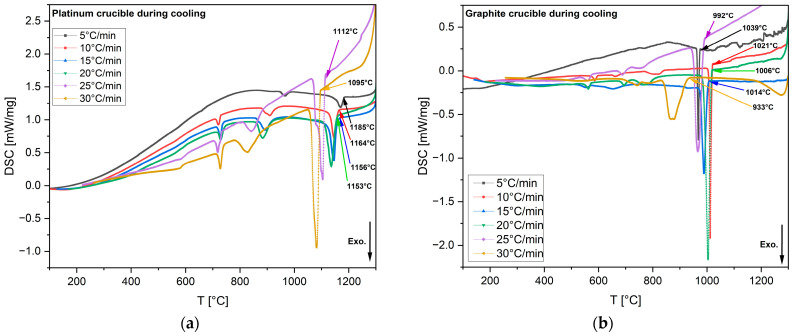
DSC heat flow curves from non-isothermal tests of mold flux specimens at various cooling rates, which were conducted in (**a**) platinum crucibles and (**b**) graphite crucibles. The legends indicate 5, 10, 15, 20, 25, and 30 °C/min cooling rates. Arrows mark the onset of primary crystallizations for each cooling rate, with arrow colors corresponding to the DSC curves. In the images above, the exothermic heat flow is directed downward.

In the DSC, changes in heat flow or calorific values were measured across various temperatures. During fusion or crystallization, the DSC curve showed an exothermic peak, indicating the release of excess heat during crystallization. This process decreased the system’s entropy, driving it to a lower energy state. Figure 5 presents the heat flow versus temperature curves from non-isothermal DSC tests performed on 60 mg mold flux specimens at various cooling rates (5, 10, 15, 20, 25, and 30 °C/min) using platinum and graphite crucibles. Crystallization temperatures were determined by the onset of exothermic peaks (shown in a downward direction in Figure 5) during cooling.

**Figure 6:**
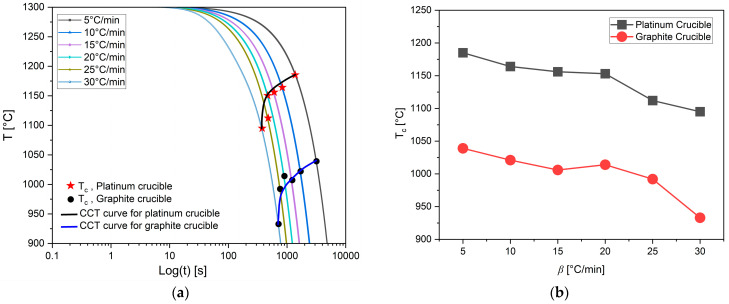
(**a**) The diagram shows the standard CCT curves of the mold flux constructed from crystallization temperatures at various cooling rates. Red stars and black circles represent the crystallization temperatures, T_c_, for all different cooling rates in platinum and graphite crucibles, respectively. The black and blue curves show CCT curves for platinum and graphite crucibles, respectively. The legend indicates the cooling rates. (**b**) The difference in the onset of primary crystallization temperatures of the mold flux at different cooling rates in platinum and graphite crucibles.

**Figure 7:**
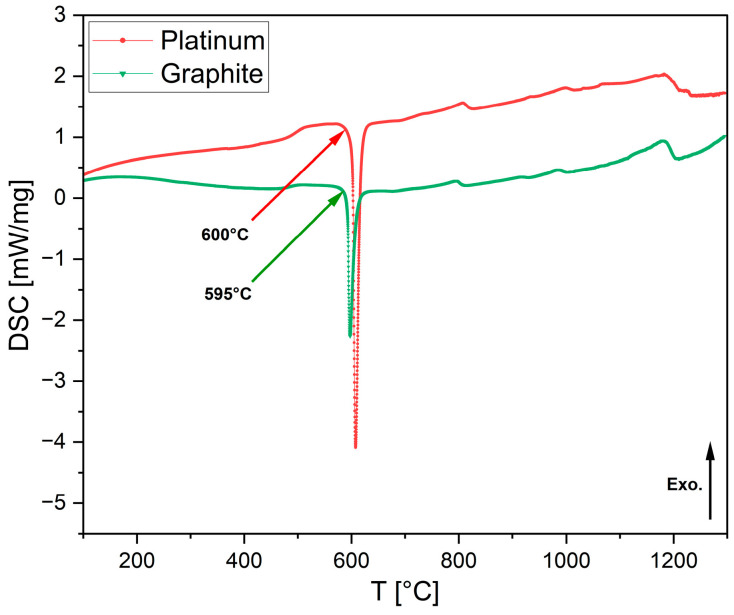
DSC heat flow curves from non-isothermal tests of mold flux specimens at 20 °C/min heating rate, which were conducted in platinum and graphite crucibles. Arrows mark the onset of mold flux melting peaks with the temperatures during heating, with arrow colors corresponding to the DSC curves. In the images above, the exothermic heat flow is directed upward.

In Equation (2), *θ* becomes small when the surface tension between the two phases is low. Complete wetting is defined as *θ* = 0. When *θ* < 90°, in the wetting condition, surface tension and free energy for nucleation increase. This was observed in mold flux tested in platinum crucibles. In contrast, in a non-wetting scenario, more undercooling is required to provide the additional free energy for nucleation. As a result, the primary crystallization temperature decreases under non-wetting conditions, such as when the mold flux was tested in graphite crucibles. All DSC experiments with mold flux in graphite crucibles showed lower primary crystallization temperatures than those with platinum crucibles, across all cooling rates. Figure 6 illustrates this phenomenon across the range of cooling rates for both crucible materials. In Figure 6a, continuous cooling transformation (CCT) curves are plotted based on the primary crystallization tested for all cooling rates in both graphite and platinum crucibles. Figure 6b shows the onset of primary crystallization shifting to lower temperatures when graphite crucibles were used. It was also observed that the exothermic peaks on the DSC curves shifted to lower temperatures, and the shape of these peaks became sharper as the cooling rate increased. A similar trend was seen for both crucibles. This can be attributed to the dependence of crystal nucleation and growth rates on viscosity and undercooling. At higher cooling rates, viscosity increases rapidly, requiring a stronger driving force to initiate mold flux nucleation [2,32]. For simplicity, only the primary crystallization temperatures of the mold flux were considered in this study, as secondary crystallization temperatures are beyond the scope of this paper. There was also insignificant difference (only 5 °C) in the mold flux melting peaks for both crucibles during the heating at a heating rate of 20 °C/min (Figure 7), which could have been due to small experimental errors. This small difference in the melting onset temperature is negligible compared to the crystallization temperature difference during cooling, which shows evidence of the effect of wetting on mold flux crystallization.

Table 3 summarizes the various onset temperatures for primary crystallization in graphite and platinum crucibles. On average, the onset of primary crystallization occurred at 143 °C lower during cooling when switching from a platinum crucible to a graphite crucible. In the case of the platinum crucible, good wettability between the liquid flux and the crucible appeared to favor the heterogeneous nucleation of the solid. As a result, increased wetting promoted crystallization. Consequently, lower onset crystallization temperatures (higher undercoolings) were observed in the presence of graphite crucibles. Table 4 presents the mass losses during all tests in both crucibles in the DSC. On average, the DSC tests showed a mass loss of approximately 3%, indicating minimal material degradation or volatilization during the experiments. Moreover, the losses were decreased with increased cooling rates for each crucible type.

### 3.3. Observation of Onset of Primary Crystallization in CLSM

**Figure 8:**
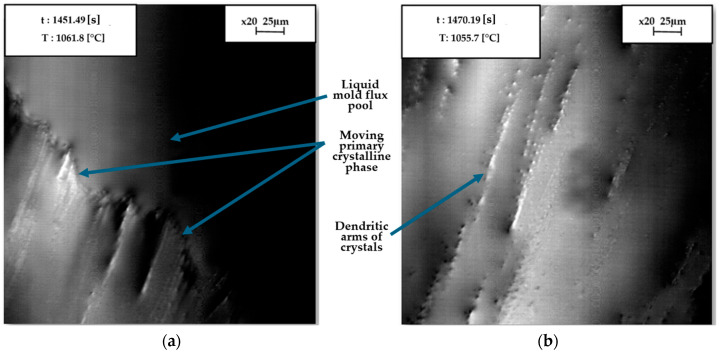
The crystallization process of the mold flux specimen in the graphite crucible was observed at a cooling rate of 20 °C/min. The images were focused on the center of the specimen surface, where the bright spot was located: (**a**) growth of the primary crystalline phase as it moved diagonally across the liquid flux; (**b**) instantaneous time of progressed undercooling, which led to crystal growth and the development of secondary dendritic arms perpendicular to the surface. t and T denote time and temperature respectively.

In situ crystal nucleation and growth on the mold flux liquid surface were observed using CLSM. A graphite crucible was used to analyze the mold flux in CLSM. Figure 8, accompanied by a scale bar for reference, presents images captured at specific times and temperatures during the crystallization process of the flux, when the temperature was decreasing from the melting point at a cooling rate of 20 °C/min. Initially, a bright spot was identified and focused on once the flux specimen became fully liquid, which occurred 2 min after homogenization at 1300 °C, following a heating rate of 20 °C/min. As the cooling continued, the edge of the crystal phase emerged from the left and expanded diagonally toward the right. The growing crystal phase became visible on the surface of the molten flux, appearing to move through the molten material. As the temperature gradually decreased, the primary crystal phase continued to grow. In Figure 8b, secondary dendritic arms are visible, forming perpendicular to the surface as the temperature decreases.

The onset of primary crystallization for the tested flux at the same cooling rate of 20 °C/min in the CLSM was slightly offset (47 °C) compared to the DSC results in the graphite crucible. Previous studies in the literature [33] indicate that the temperature difference between the specimen surface and the bottom of the crucible (where the thermocouple is positioned) can be as much as 40 °C in CLSM, which is consistent with the findings of this study. The confocal microscope uses spatial filtering to create a focused illumination spot. As a result, crystallization can only be visualized at the focused spot in CLSM [1]. Therefore, the quantification of crystallization temperature in CLSM is less precise than in DSC. Using CLSM, locating the nucleation point is challenging; however, crystal growth can be visualized. Furthermore, nucleation appeared to occur on the crucible wall, with crystals migrating from one side of the crucible in CLSM, as shown in Figure 8.

These crystals apparently nucleate at low temperatures on the crucible wall, and it is not possible to directly measure the temperature of the crucible wall. Instead, reliance must be placed on the system’s overall temperature, which is detected at the bottom of the crucible by the thermocouples in CLSM. Nevertheless, the onset of primary crystallization temperature in the graphite crucible found in CLSM was significantly lower than that found in the DSC test in the platinum crucible under the same conditions. This phenomenon is consistent with the observation that non-isothermal crystallization tests of mold flux in a non-wetting graphite crucible resulted in delayed crystal precipitation during cooling. This delay was attributed to the need for additional energy to overcome the nucleation barrier, facilitated by greater undercooling.

### 3.4. Identification of Primary Crystal Phase in XRD and SEM-EDS

**Figure 9:**
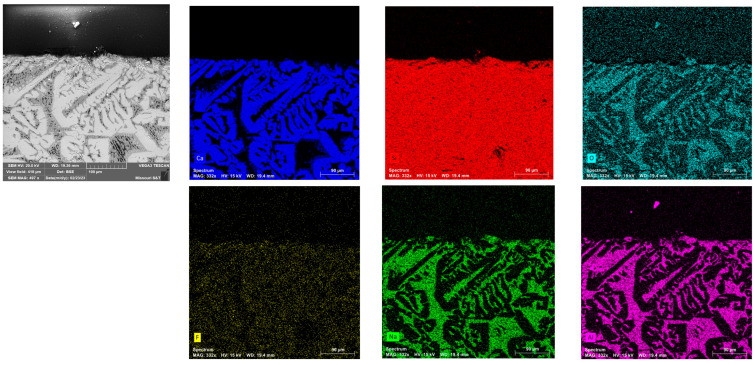
The backscattered electron (BSE) SEM image of the polished and coated mold flux specimen is shown in the **top**-**left** corner, along with images generated from elemental maps using SEM-EDS. The black region at the top of the images represents the epoxy area.

The flux specimen, quenched in CLSM after primary crystal formation, was further analyzed using SEM-EDS. The post-experiment CLSM mold flux specimen in the graphite crucible was found to form into a spherical shape due to non-wetting interactions with the graphite wall. As a result, the specimen was easily recovered from the crucible. Microstructural analysis of the flux specimen, combined with EDS (Figure 9), revealed the presence of primary cuspidine crystals (Ca_4_Si_2_O_7_F_2_) dispersed within a glassy matrix. Elemental mapping of the observed area shows that Ca, Si, O, and F are concentrated in the white regions of the BSE image, while Na, Al, Si, and O are concentrated in the gray areas. This illustrates the distribution of the cuspidine crystalline phase within the glassy matrix. Since the primary crystals nucleated and grew at a slow cooling rate (5 °C/min) from 1300 °C to 1000 °C before rapid cooling, the morphology of the cuspidine crystals was found to be faceted, which was consistent with findings from a previous study [2].

**Figure 10:**
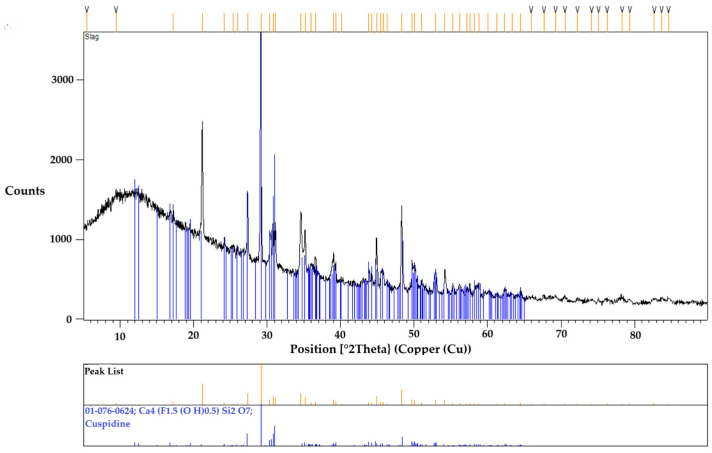
The XRD result of the mold flux specimen shows cuspidine as the primary crystal phase.

To confirm the accuracy of the obtained results, another specimen underwent the same thermal cycle and was then crushed into powder for XRD analysis. This SEM-EDS observation was consistent with the XRD results, as shown in Figure 10, which identified cuspidine as the primary and only crystalline phase. The absence of any other phases in the XRD analysis can be attributed to its glassy matrix appearance in the specimen. Although the XRD results showed distinct diffraction peaks for cuspidine, a prominent amorphous hump was initially observed at low angles. This hump is characteristic of a glassy phase, likely due to the significant presence of a glassy matrix. The SEM-EDS analysis, mentioned earlier, also identified the elements that seems to be in the glassy matrix.

### 3.5. Sensitivity of Graphite Crucible in DSC

**Figure 11:**
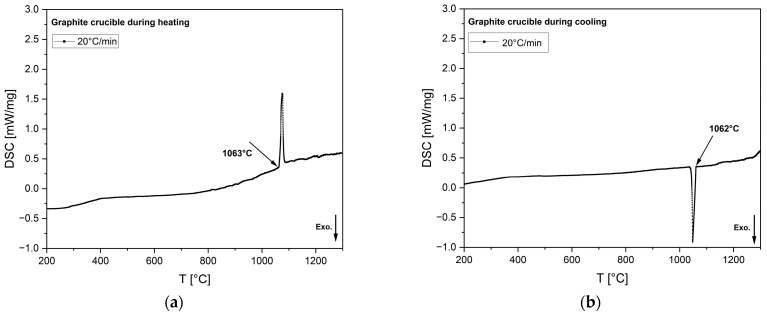
DSC heat flow curves of gold tested in graphite crucible during (**a**) heating and (**b**) cooling at a 20 °C/min heating/cooling rate. Arrows indicate the onset of solidification or phase transformation, corresponding to the temperature. In the images above, the exothermic heat flow is directed downward.

To verify its sensitivity, the graphite crucible was calibrated in the DSC using a gold standard at a moderate cooling rate of 20 °C/min. The calibrated DSC curves for both heating and cooling are shown in Figure 11.

The standard phase transition temperature of gold is 1064 °C. Gold tested in the graphite crucible displayed good agreement with the expected standard transformation, with deviations of only 1 °C during heating and 2 °C during cooling, which verifies the test equipment and methodology.

Notably, there is a possibility of a reaction between the transition metal oxides in the mold flux and the graphite crucible. To eliminate this factor, a synthetic slag was tested in the DSC (20 °C/min cooling rate) using both graphite and platinum crucibles. Table 5 presents the chemical composition of the slag, which is devoid of transition metal oxides such as Fe_2_O_3_ and MnO.

**Figure 12:**
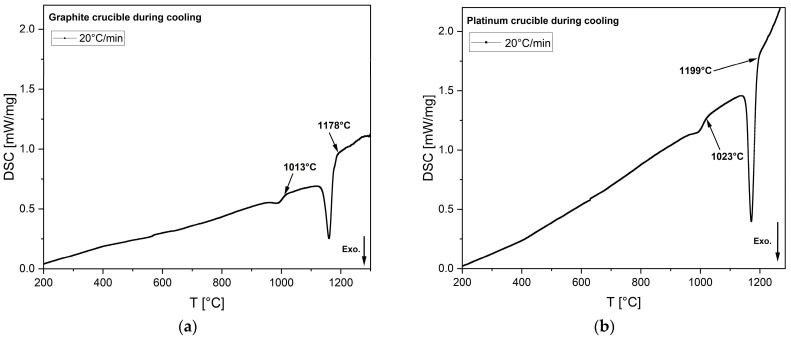
DSC heat flow curves of synthetic slags tested in (**a**) a graphite crucible and (**b**) a platinum crucible at a cooling rate of 20 °C/min. Arrows indicate the onset of solidification or phase transformation corresponding to the temperature. In the above pictures, the direction of exothermic heat flow is downward.

A higher-basicity synthetic slag that readily crystallizes during cooling was selected for the experiment. Notably, the onset of primary crystallization occurred at 21 °C lower in the graphite crucible than the platinum crucible, despite the synthetic slag containing no transition metal oxides. This observation supports the notion that the wetting characteristics between the slag and the inner wall of the platinum crucible promote nucleation and that the differences in crystallization temparature are not the result of reactions with the crucible. This experiment required an additional 21 °C of undercooling for the initial phase transition. Figure 12 illustrates the exothermic peaks of phase transitions during the cooling of synthetic slags in both graphite and platinum crucibles, tested at a cooling rate of 20 °C/min.

**Figure 13:**
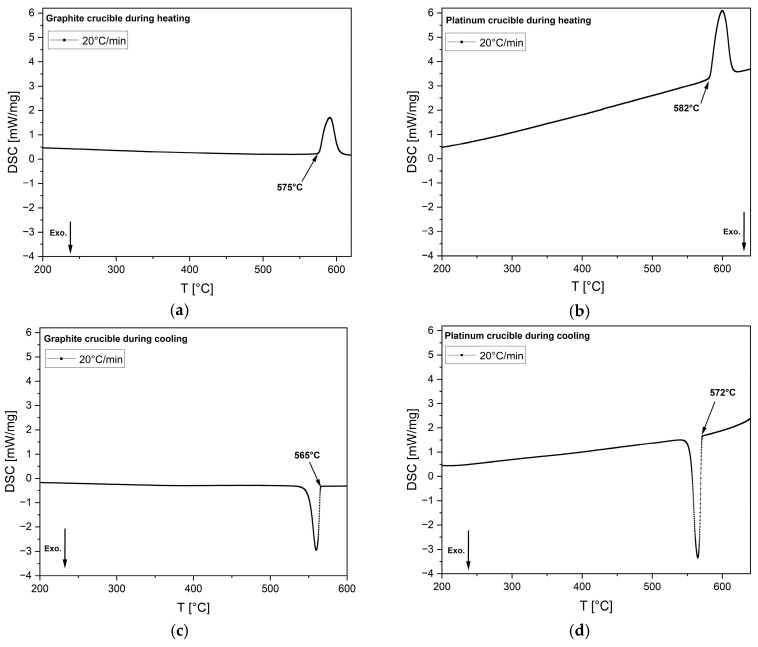
DSC heat flow curves of the standard material Li_2_SO_4_ tested under the following conditions: (**a**) in a graphite crucible at a heating rate of 20 °C/min; (**b**) in a platinum crucible at a heating rate of 20 °C/min; (**c**) in a graphite crucible at a cooling rate of 20 °C/min; and (**d**) in a platinum crucible at a cooling rate of 20 °C/min. Arrows mark the onset of solidification or phase transformation at the corresponding temperatures. In the images above, exothermic heat flow is directed downward.

**Figure 14:**
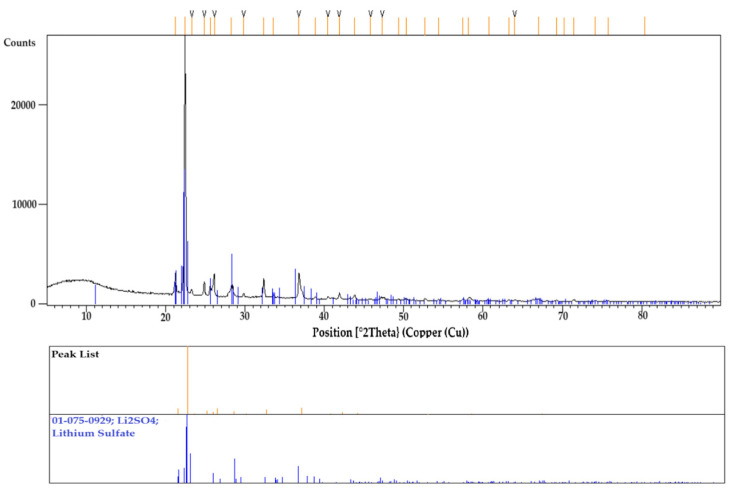
XRD result of Li_2_SO_4_ specimen showing the only phase of lithium sulfate tested in the graphite crucible.

Li_2_SO_4_ is the only standard calibration material compatible with both graphite and platinum crucibles. Consequently, it was tested in the DSC at a cooling rate of 20 °C/min using both crucible types to measure the phase transformation temperature, as shown in Figure 13. The melting temperatures during heating were found to be very similar: −3 °C for the graphite crucible and +4 °C for the platinum crucible, relative to the standard melting temperature of Li_2_SO_4_ (578 °C). During cooling, the phase transformation temperatures were also comparable, measuring 572 °C for the platinum crucible and 565 °C for the graphite crucible. The −6 °C temperature deviation observed in the platinum crucible could be attributed to operational error, whereas the −13 °C offset for the graphite crucible is likely due to a more pronounced non-wetting condition. This condition may necessitate greater undercooling to supply the additional free energy required for nucleation to occur. After the DSC experiment, the Li_2_SO_4_ specimen tested in the graphite crucible was collected and ground into powder for XRD analysis. To remove any moisture content, the specimen was preheated prior to analyzing. The diffraction peaks confirmed the presence of only Li_2_SO_4_, with no additional phases observed from the ICSD. Figure 14 presents the XRD results.

### 3.6. Thermodynamic Equilibrium Simulations of Li_2_SO_4_ with Carbon and Platinum

**Figure 15:**
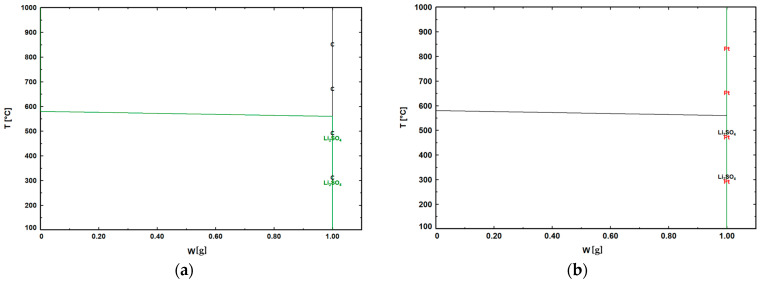
Phase evolution in equilibrium for the following reactions: (**a**) C with Li_2_SO_4_; and (**b**) Pt. with Li_2_SO_4_, as determined through thermodynamic calculations.

Potential interfacial reactions between Li_2_SO_4_ and either platinum or graphite were investigated using thermodynamic equilibrium simulations. Figure 15 presents the phase stability diagrams for reactions involving 1 g of carbon (C) or platinum (Pt.) with Li_2_SO_4_ over the temperature range of 1000 °C to 0 °C, generated using FactSage^®^. In both cases, Li_2_SO_4_ solidified at 578 °C without the formation of any additional phases. The experimental results were in close agreement with the thermodynamic simulation predictions. Therefore, the observed reduction in the solidification temperature of Li_2_SO_4_ in the graphite crucible can be attributed solely to its wetting behavior.

### 3.7. Detection of Potential Interactions at the Interface of Flux and Graphite Crucible in SEM-EDS

After the DSC experiment, the mold flux specimen tested in the graphite crucible at a cooling rate of 20 °C/min was recovered and mounted in epoxy to enable grinding and polishing. The specimen was coated with an Au-Pd layer and subjected to SEM-EDS analysis to investigate potential interfacial reactions. Prior to testing, the mold flux specimens were calcined to eliminate any carbon content in the flux.

**Figure 16:**
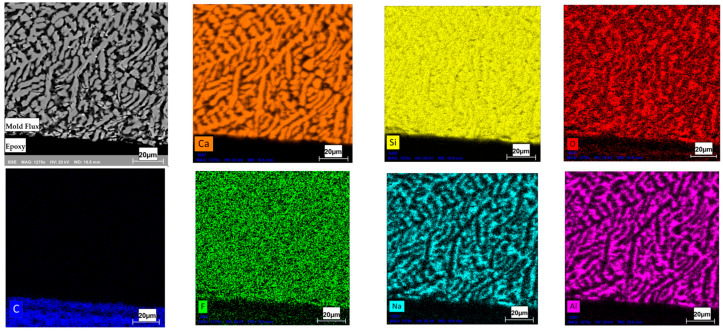
The backscattered electron (BSE) SEM image of the polished and coated mold flux specimen (top-left corner) alongside elemental maps generated by SEM-EDS. The black area in the top-left image corresponds to the epoxy region. No traces of carbon were detected within the mold flux, except in the epoxy region, and no reaction layers were observed at the interface. The cuspidine crystals (Ca_4_Si_2_O_7_F_2_) dispersed within a glassy matrix (consists of Na, Al, Si, and O) in the mold flux specimen as explained earlier.

Figure 16 presents the elemental maps obtained through SEM-EDS from a cross-section of the flux specimen. The image was captured to include a portion of the epoxy for reference. The EDS analysis confirmed that no appreciable carbon was detected within the mold flux and no reaction layers were observed at the flux–crucible interface. This indicates no reactions likely occurred between the mold flux and the graphite crucible’s inner wall during the DSC experiments and that no chemical reactions between the flux specimen and the graphite crucible occurred. This analysis clarified that the observed variations in crystallization behavior were due to physical wetting effects rather than chemical interactions, highlighting the role of the crucible material in influencing crystallization.

### 3.8. Wetting Conditions on Crystallization

Mold flux nucleation or cuspidine crystallization is dominated by solid wetting in the liquid–solid–substrate system. On the other hand, there is a gas–liquid–mold interface in the vicinity of the mold wall side in the mold gap. The wetting of the liquid to the crucibles in DSC and CLSM does not necessarily indicate that the solid also wets the crucible material. In these experiments, cuspidine appeared to be preferentially nucleated under liquid-wetting conditions, suggesting that the solid also wets the substrate as well. Therefore, there appears to be some correspondence between the solid wettability and the inherent liquid wettability to lower the overall interfacial energy.

The present study suggests that the occurrence of the crystallization at the crucible wall, i.e., the wettability of solid cuspidine to the crucible, is enhanced when the liquid flux wets the platinum crucible. For cuspidine crystallization in a graphite crucible, the nucleation is suppressed to a low temperature, suggesting that the solid–liquid wetting angle for cuspidine in graphite is higher and that nucleation is suppressed.

The wetting condition of the mold flux affects crystallization behavior, and as a result, it may also affect heat removal and lubrication in the mold gap. Hence, it may impact the cast product’s final quality. Moreover, if the mold wall exhibits a non-wetting condition with devitrified cuspidine crystals in the mold gap, the crystallization temperature will drop significantly below the expected level due to the lack of wettability. Consequently, the design criteria for the physical properties of the mold flux will be altered, potentially compromising its effectiveness in facilitating the efficient casting of the final products.

## 4. Conclusions

The effect of mold flux wetting conditions with varying crucible materials on crystallization was conducted in this article, and the following conclusions can be drawn:

1. The conventional CaO-SiO_2_-based mold flux exhibits strong wetting behavior with platinum crucibles but demonstrates a non-wetting condition when in contact with graphite crucibles.
2. In all non-isothermal DSC experiments, mold flux specimens in graphite crucibles consistently showed significantly lower primary crystallization temperatures compared to those in platinum crucibles across all tested cooling rates (5, 10, 15, 20, 25, and 30 °C/min). The tendency for heterogeneous nucleation is suppressed in a system that is non-wetting (graphite), compared to a system that is wetting (platinum).
3. In situ solidification tests of mold flux in graphite crucibles using CLSM also revealed a decrease in the onset of primary crystallization temperature.
4. Temperature measurements in the DSC with graphite and platinum crucibles using gold and Li_2_SO_4_ standards were found to be accurate to within a few degrees. DSC tests on synthetic slag exhibited a similar trend to mold flux, finding lower crystallization temperatures in graphite crucibles compared to platinum crucibles.
5. The SEM-EDS analysis of the flux specimen in the graphite crucible confirmed that no reactions occurred between the mold flux and the graphite crucible during the DSC experiments, clarifying that observed variations in crystallization behavior were due to the wetting characteristics of the crucible material rather than chemical interactions.
6. Overall, the wetting condition of the mold flux promotes primary crystallization, while its non-wetting characteristic suppresses nucleation.
7. In the future, efforts will be made to implement a molten tin “quench and age” technique to study mold flux behavior in graphite and platinum crucibles over time. Aged specimens will be analyzed by using quantitative XRD and SEM to characterize primary and secondary crystallites and study their structure, nucleation, and growth mechanisms. This will enhance the understanding of mold flux crystallization, particularly the influence of crucible material on crystal phase formation, and their kinetics. Most importantly, it will facilitate the crucible materials’ effect analysis on primary and secondary crystals’ nucleation and growth, to understand better the practical mold gap in the continuous casting process.

## Figures and Tables

**Table 1:** Chemical composition [wt.%] of the mold flux that was used in this study.

CaO	SiO_2_	Na_2_O	F	Al_2_O_3_	MnO	Li_2_O	Fe_2_O_3_	MgO	C (Total)
31.7	27.5	9.2	7.6	5.2	2.8	0.7	0.5	0.3	9.4

**Table 2:** Physical properties of the mold flux.

Basicity (CaO/SiO_2_)	Solidification Temperature [°C]	Viscosity [Poise] at 1300 °C
1.15	1130	0.7

**Table 3:** The onset of primary crystallization temperatures in graphite and platinum crucibles at various cooling rates.

Cooling Rates [°C/min]	Primary Crystallization Temperatures [°C]		Crystallization Temperature Difference [°C]
5	1185	1039	146
10	1164	1021	143
15	1156	1006	150
20	1153	1014	139
25	1112	992	120
30	1095	933	162
Tested crucible	Platinum	Graphite	Average: 143

**Table 4:** Mass loss during all tests in both crucibles at various cooling rates.

Cooling Rates [°C/min]	Mass Loss During the Test [%]	
5	5	5
10	3	4
15	2.5	4
20	2.5	2
25	2.5	3
30	2	2
Tested crucible	Platinum	Graphite

**Table 5:** Chemical composition [wt.%] of the synthetic slag that was used in this study.

Specimen ID	CaO	SiO_2_	Al_2_O_3_	Na_2_O	F	Basicity
SA-3	39.3	34.1	10	7.5	9.1	1.15

## Data Availability

The original contributions presented in this study are included in the article. Further inquiries can be directed to the corresponding author.

## Abbreviations, Symbols and Notations

The following abbreviations, symbols and notations are used in this manuscript:

DSC	Differential scanning calorimetry
CLSM	Confocal laser scanning microscopy
SEM-EDS	Scanning electron microscopy coupled with energy dispersive X-ray spectroscopy
XRD	X-ray diffraction
CDMS	Copper disc mold simulator
TGA	Thermal gravimetric analyzer
BSE	Backscattered electron
ICSD	Inorganic crystal structure database
CCT	Continuous cooling transformation
C	Carbon
Pt.	Platinum
Au	Gold
Pd	Palladium
°C	Degree Celsius
°C/min	Degree Celsius per minute
s	Second
t	Time
T	Temperature
Δ*G_heterogeneous_*	Free energy for heterogeneous nucleation
Δ*G_homogeneous_*	Free energy for homogeneous nucleation
*f*(*θ*)	Function of the contact/wetting angle
*θ*	Contact/wetting angle
γ	Surface tension
*G–M*	Gas–mold interface
*G–L*	Gas–liquid interface
*L–M*	Liquid–mold interface
T_c_	Onset of primary crystallization temperature
*β*	Cooling rate
W	Weight
g	Gram
